# Bias by gender: exploring gender-based differences in the endorsement of ADHD symptoms and impairment among adult patients

**DOI:** 10.3389/fgwh.2025.1549028

**Published:** 2025-03-20

**Authors:** Noemi M. Platania, Daniëlle E. J. Starreveld, Dora Wynchank, Aartjan T. F. Beekman, Sandra Kooij

**Affiliations:** ^1^ Expertise Center Adult ADHD, PsyQ, The Hague, Netherlands; ^2^ Amsterdam UMC, VU Medical Centre, Department of Psychiatry, Amsterdam, Netherlands

**Keywords:** adult ADHD, gender differences, diagnostic tools, DIVA-5, symptom endorsement, impairment

## Abstract

**Background:**

Research on adult attention-deficit/hyperactivity disorder (ADHD) remains limited, particularly regarding the experiences of women.

**Methods:**

This exploratory study investigates patient responses to the Diagnostic Interview for ADHD in Adults (DIVA-5), which assesses current (adult) and retrospective (childhood) ADHD symptoms based on criteria from the Diagnostic and Statistical Manual of Mental Disorders (DSM-5). We focused on overall endorsement rates of ADHD symptoms, impairments, and specific examples of both, with particular attention to gender differences. Using descriptive statistics and chi-square tests, we analysed existing DIVA-5 data from 2,257 adult patients diagnosed with ADHD at mental health clinics affiliated with the Parnassia Groep in the Netherlands.

**Results:**

Our findings indicate that ADHD manifests similarly across men and women, though subtle differences in symptom and impairment patterns emerged. Women more frequently endorsed several inattentive and hyperactive/impulsive symptoms in adulthood, whereas men reported higher endorsement rates of several childhood symptoms. Regarding impairments, gender-specific patterns were observed in areas such as self-esteem and social relationships.

**Conclusion:**

While these differences were small, they highlight the need for further investigation into gendered ADHD manifestations. Additionally, we discuss potential measurement limitations and propose recommendations for refining the DIVA-5 and advancing research on gender differences in ADHD.

## 1 Introduction

Attention-deficit/hyperactivity disorder (ADHD) is typically conceptualised as a neurodevelopmental condition that affects people across their lifespan, manifesting through symptoms of inattention, hyperactivity, and impulsivity. The prevalence of ADHD is estimated to be approximately 7% in children (1) and 2.5% in adults (2). Among those diagnosed in childhood, about 57% continue to meet the diagnostic criteria for ADHD in adulthood (3). Despite its prevalence, adult ADHD remains underrepresented in research, particularly compared to childhood ADHD, leaving significant gaps in understanding how symptoms evolve with age and how they differ across subgroups (4). The consequences of living with ADHD can be severe: adults with ADHD are more likely to experience adverse outcomes such as early termination of relationships or jobs (5), increased risk of cardiovascular disease (6), and higher mortality rates (7). Early diagnosis can mitigate such adverse outcomes by ensuring timely intervention, potentially reducing preventable suffering and dysfunction [see e.g., (8)].

A key issue in ADHD diagnosis is that women may be overlooked during the diagnostic process in childhood and adolescence. Women tend to be diagnosed later than men; for example, a retrospective observational study of four US health databases found that the mean age of ADHD diagnosis ranged from 16.3 to 28.6 years for females, compared to 11.2 to 22.7 years for males (9). Although there are many potential interacting reasons for this disparity [see e.g., (10)], one possibility is that our understanding of female ADHD is limited because it was long considered a “male” condition. Early research predominantly focused on males, thereby potentially overlooking the specific characteristics of ADHD in females (11). For instance, in the field trials conducted to establish the diagnostic criteria for ADHD, as outlined in the Diagnostic and Statistical Manual of Mental Disorders (DSM-5) and the International Classification of Diseases (ICD-11), only 21% of participants were girls (12). It is hence likely that many diagnostic tools were not designed with women and girls in mind, and that our understanding of ADHD in women is limited.

The Diagnostic Interview for ADHD in Adults (DIVA-5) is a semi-structured tool used worldwide to diagnose ADHD in adults (13). It assesses the eighteen diagnostic criteria for ADHD outlined in the DSM-5, as well as several functional impairment categories, through the use of specific examples of behaviours. For instance, the criterion for “Forgetfulness” includes examples such as “Forgets keys, agenda, etc.”. Each criterion is accompanied by examples from both childhood and adulthood, aiding clinicians in determining whether the criterion is met. There are no studies to date that have examined how frequently patients select these behavioural examples during assessment. Exploring these patterns could be valuable for several reasons. Firstly, it might provide useful information on the manifestation of ADHD in adults across genders, shedding light on the nuances of symptomatology that may go unnoticed in more general analyses of diagnostic criteria. Secondly, examining potential gender differences in example selection frequencies per DSM-5 criterion could enhance our understanding of whether and how ADHD manifests differently in women vs. men and identify any biases in the DIVA-5 itself. Finally, such insights could inform improvements to the DIVA-5.

In addition to examining example selection in the DIVA-5, investigating ADHD symptom endorsement overall and across genders could help us to better understand adult ADHD. This is especially important given the phenotypic variation of the disorder. Since adults require five or more out of nine inattentive symptoms and/or five or more hyperactive/impulsive symptoms to meet the diagnostic threshold according to the DSM-5, there are many possible combinations of symptoms that any given patient might experience. Additionally, research has shown that individual symptoms contribute uniquely to the clinical diagnosis, with some carrying more diagnostic weight than others (14). Therefore, relying solely on sum scores to assess ADHD might oversimplify the complexity of the disorder and obscure important clinical nuances. For instance, Biederman et al. (15) found that stimulant medication treatment in young adults was associated with improved performance for some neuropsychological functions but not others, suggesting the impact of treatment might vary by symptom. Understanding patterns in symptom endorsement frequencies, particularly across gender, could therefore provide insights for refining diagnostic approaches and developing more tailored treatments for adult ADHD.

Previous studies have examined the endorsement of the DSM criteria for ADHD in adults, with the main finding being that inattention symptoms are more commonly endorsed than hyperactivity/impulsivity symptoms [see e.g., (16–19)]. However, these studies are based on the criteria from the DSM-IV, and the transition to DSM-5 introduced changes in symptom descriptions that warrant a re-examination using DSM-5 data (20). To our knowledge, only three studies have specifically examined DSM-5 symptom endorsement in adults (20–22). Although these studies generally found similar trends—reporting “Easily distracted” as the most endorsed inattention symptom and “Fidgets” as the most endorsed hyperactivity/impulsivity symptom—their findings differ widely in terms of overall endorsement rates. The studies are also limited by relatively small sample sizes and exclusively young adult populations. A larger, more diverse study using the DIVA-5, which directly aligns with DSM-5 criteria, could therefore offer additional valuable insights into adult ADHD symptom presentation and potential gender differences.

Another area of adult ADHD that warrants investigation is impairment due to ADHD symptoms. The DSM-5 requires clear evidence that ADHD symptoms affect social, school, or work functioning as a criterion for diagnosis. Although ADHD-related impairments are linked to symptomatology, research shows that the relationship between symptom severity and impairment is only modest (23). Despite this, much of the existing research focuses on ADHD symptoms, often overlooking the broader impact of impairment on daily life. Studies have found that adults with ADHD experience the greatest impairments in work performance and interpersonal relationships (5, 24, 55), with men more impaired in work and women more impaired socially (56). While these insights are valuable, a more comprehensive exploration of impairment across multiple domains is needed to better understand the impact of adult ADHD. The DIVA-5 is particularly suited for this, as it directly assesses ADHD-related impairments across five domains, each with concrete examples. Investigating which areas of functioning are most and least commonly affected in adults with ADHD could offer a more nuanced understanding of the disorder's impact on men and women. Moreover, examining selection frequencies of the examples within each impairment category could reveal how impairment manifests and whether gender plays a role. Such findings might eventually improve diagnostic practices and lead to more tailored interventions targeting the most disabling aspects of ADHD.

In this study, we analyse existing patient responses to the DIVA-5 from a large sample of patients treated at mental health clinics of the Parnassia Groep in the Netherlands. We investigate the frequency of example selection for each ADHD symptom and impairment category, as well as the overall endorsement of symptoms and impairments. Additionally, we explore potential gender differences in these frequencies. Given the relative novelty of this study, we did not formulate any *a priori* hypotheses. Our primary goal is to report on the collected data in an exploratory way.

## 2 Methods

### 2.1 Study design and participants

This study uses existing patient data collected from the Parnassia Groep, a mental healthcare institution operating across various regions in the Netherlands. The sample consists of all adult patients diagnosed with ADHD who completed the DIVA-5 as part of their diagnostic evaluation and treatment at Parnassia Groep locations between June 2021 and January 2024. We chose June 2021 as the starting point to mitigate the potentially confounding effects of the COVID-19 pandemic, as most heavy restrictions in the Netherlands were lifted at that time. Patients were referred by a general practitioner or mental health professional, following the standard procedure for mental health treatment in the Netherlands. The sample represents a broad demographic range, as patients attended various clinics throughout the country. For patients with multiple DIVA-5 assessments, only the initial one was included in the analysis.

The study was conducted in accordance with the Declaration of Helsinki. To protect patient privacy, the data were de-identified before we received them. Due to the retrospective nature of the study and the de-identification of data, the requirement for informed consent was waived. The medical research ethics committee of Amsterdam University Medical Center reviewed the study protocol and determined that ethical approval was not required (No. 2024.0509). This decision was based on the fact that patients were not subjected to any additional procedures beyond their standard treatment.

### 2.2 Measures

#### 2.2.1 Demographic measures

The demographic data collected included age at the time of the DIVA-5 assessment and legal gender (male or female), obtained during patient registration based on information provided by referring clinicians.

#### 2.2.2 DIVA-5

The DIVA-5 is a semi-structured interview which has been translated into more than thirty languages and is used to diagnose ADHD in adults around the world. It is structured into three sections, each applied to both adulthood (from age seventeen) and childhood (before age twelve): (1) criteria for inattention, (2) criteria for hyperactivity/impulsivity, and (3) impairment caused by the symptoms and symptom onset. In addition to this adult version, adaptations include the Young DIVA-5 for children and adolescents and the DIVA-5 ID for people with intellectual disabilities.

The symptoms of ADHD are addressed in the first two sections of the interview. For each symptom, clinicians initially prompt patients to provide their own examples of related behaviours, evaluating whether they occur frequently and have persisted for at least six months. If necessary, clinicians can then offer examples from a predefined list and ask if the patient identifies with them. These examples were originally designed based on clinical expertise. Clinicians mark each example that resonates with the patient. Additional examples can be recorded in an open text field, but these open responses are not analysed in this study. Section 3 assesses impairment across five life areas, such as work/education and relationships, using the same example-based process. Clinicians also evaluate both ADHD symptom severity and subtype. Whenever possible, the DIVA-5 is completed in the presence of an informant, such as a partner and/or family member, for additional information about symptoms and impairment.

In this study, trained psychologists and psychiatrists at Parnassia Groep locations administered the Dutch version of the DIVA-5. The translations used in this paper are based on the English version of the DIVA-5.

### 2.3 Statistical analysis

We began by computing descriptive statistics to characterise the sample in terms of age, ADHD subtype, and ADHD severity. To compare these variables across gender, we used an independent *t*-test for age and Pearson's *χ*² tests for ADHD subtype and severity.

Next, we analysed the DIVA-5 responses. We calculated the proportion of patients endorsing each example provided in the interview and the proportion of patients meeting the diagnostic criteria for each ADHD criterion and impairment category, as assessed by the clinician. These outcomes were assessed twice: once for current symptoms and once for childhood symptoms (before the age of twelve). The proportions were calculated for the overall sample and separately for men and women. To compare the frequencies across genders, we used Pearson's *χ*^2^ tests and calculated the φ (phi) coefficient to measure the effect sizes. We also derived odds ratios, including their standard errors and confidence intervals, to assess the strength of associations; these are presented in the Supplementary Material for reference.

An alpha level of.05 was set for all tests, without multiplicity adjustment, as recommended by Rubin (25). All analyses were performed using R version 4.2.1 and MS Excel version 2,402. The R scripts are publicly available online (see OSF: https://osf.io/t6hp7/), and the data can be provided upon request.

## 3 Results

### 3.1 Sample characteristics

The demographic and clinical characteristics of 2,257 patients are presented in Table 1. Approximately 61% of the sample was classified as female. Male and female participants did not differ in their distribution of ADHD subtype and severity. However, female participants were slightly younger compared to male participants (*p* < .001). Most participants completed the DIVA-5 in the presence of a family member or partner, except for 257 who completed it alone.

**Table 1:** Patient demographic and clinical characteristics, by gender (*n* = 2,257 for age; *n* = 2,129 for ADHD subtypes and severity).

	Males	Females	Total sample
	*n* (%)	*n* (%)	*n* (%)
Mean age (*SD*)*	34.40 (11.02)	32.07 (11.00)	33.38 (11.09)
Total	881 (39.03)	1,376 (60.97)	2,257 (100.00)
ADHD subtypes			
Combined presentation	525 (59.59)	832 (60.46)	1,357 (60.12)
Predominantly inattentive presentation	289 (32.80)	420 (30.52)	709 (31.41)
Predominantly hyperactive/impulsive presentation	12 (1.36)	32 (2.33)	44 (1.95)
Other specified ADHD	1 (0.11)	10 (0.73)	11 (0.49)
Unspecified ADHD	2 (0.23)	6 (0.44)	8 (0.35)
Total	829 (94.10)	1,300 (94.48)	2,129 (94.33)
ADHD severity			
Mild	120 (13.62)	217 (15.77)	337 (14.93)
Moderate	612 (69.47)	963 (69.99)	1,575 (69.78)
Severe	97 (11.01)	120 (8.72)	217 (9.61)
Total	829 (94.10)	1,300 (94.48)	2,129 (94.33)

* *p* < .001.

### 3.2 Missing data

There were no missing data for the overall symptom and impairment category analysis. For the symptom and impairment examples, approximately 1% of data points were missing. Given the small proportion, we opted to exclude these missing data points from the relevant analyses to maintain data integrity and simplify the analysis process. Data on ADHD severity and subtype were incomplete for about 6% of patients; as such, percentages reported for these variables are based on the available data.

### 3.3 Adulthood symptoms

The endorsement rates for adulthood ADHD symptoms by gender are presented in Table 2. Overall, symptoms were frequently endorsed, with “Difficulty sustaining attention” being the most common, reported by over 97% of all patients (full-sample endorsement percentages are available in the Supplementary Material). Even the least endorsed symptom, “Interrupts or intrudes on others”, was still reported by more than 40% of patients. Inattention symptoms were generally more commonly endorsed than hyperactivity/impulsivity symptoms.

**Table 2:** Percentage of ADHD symptoms endorsed, by gender (*n* = 2,257).

	Adulthood symptoms					Childhood symptoms				
	Males	Females	*χ* ^2^	φ	OR (95% CI)	Males	Females	χ^2^	φ	OR (95% CI)
Inattention										
Fails to pay close attention to details/makes careless mistakes	81.16	80.45	0.130	0.01	1.05 (0.84–1.30)	68.22	61.34	10.74**	0.07	1.35 (1.13–1.62)
Has difficulty sustaining attention	97.39	97.38	0.00	0.00	1.00 (0.59–1.70)	85.58	82.78	2.93	0.04	1.24 (0.98–1.56)
Does not seem to listen when spoken to directly	80.14	82.48	1.82	0.03	0.86 (0.69–1.06)	67.88	63.23	4.91*	0.05	1.23 (1.03–1.47)
Does not follow through on instructions, fails to finish chores/duties	88.31	92.66	11.84***	0.07	0.60 (0.45–0.80)	75.60	69.48	9.65**	0.07	1.36 (1.12–1.65)
Has difficulty organising tasks and activities	85.36	89.97	10.52**	0.07	0.65 (0.50–0.84)	70.60	73.18	1.66	0.03	0.88 (0.73–1.06)
Avoids, dislikes, or is reluctant to engage in tasks that require sustained mental effort	91.15	91.71	0.16	0.01	0.93 (0.69–1.26)	78.09	70.42	15.81***	0.08	1.50 (1.23–1.82)
Loses things necessary for tasks or activities	71.40	80.38	23.87***	0.10	0.61 (0.50–0.74)	52.10	56.10	3.31	0.04	0.85 (0.72–1.01)
Is easily distracted by extraneous stimuli	92.28	93.89	1.98	0.03	0.78 (0.56–1.08)	83.65	81.32	1.84	0.03	1.18 (0.94–1.47)
Is forgetful in daily activities	77.30	81.39	5.34*	0.05	0.78 (0.63–0.96)	57.89	53.71	3.63	0.04	1.18 (1.00–1.41)
Hyperactivity/impulsivity										
Fidgets with hands or feet or squirms in seat	81.72	80.38	0.55	0.02	1.09 (0.88–1.36)	71.51	66.35	6.37*	0.05	1.27 (1.06–1.53)
Leaves seat in classroom/in other situations in which remaining seated is expected	52.67	49.85	1.59	0.03	1.12 (0.94–1.33)	45.97	34.96	26.86***	0.11	1.58 (1.33–1.88)
Feels restless	78.55	87.72	33.16***	0.12	0.51 (0.41–0.64)	54.48	47.67	9.69**	0.07	1.31 (1.11–1.56)
Has difficulty engaging in leisure activities quietly	40.41	47.31	10.08**	0.07	0.75 (0.64–0.90)	39.61	37.35	1.07	0.02	1.10 (0.92–1.31)
Is often on the go or often acts as if “driven by a motor”	63.79	72.60	19.14***	0.09	0.66 (0.55–0.80)	54.37	54.43	0.00	0.00	1.00 (0.84–1.18)
Talks excessively	52.44	62.79	23.32***	0.10	0.65 (0.55–0.78)	42.45	54.94	33.02***	0.12	0.60 (0.51–0.72)
Blurts out answers before questions have been completed	65.61	70.57	5.91*	0.05	0.79 (0.66–0.95)	44.95	49.85	4.98*	0.05	0.82 (0.69–0.97)
Has difficulty awaiting turn	57.89	60.46	1.37	0.02	0.90 (0.76–1.07)	48.58	46.88	0.56	0.02	1.07 (0.90–1.27)
Interrupts or intrudes on others	40.29	44.62	3.93*	0.04	0.84 (0.71–0.99)	37.12	38.08	0.17	0.01	0.96 (0.81–1.14)

* *p* < .05; **p < .01; ***p < .001.

OR = Odds Ratio. OR > 1 indicates higher odds in males; OR < 1 indicates higher odds in females. The 95% Confidence Interval (CI) shows the range within which the true OR likely falls.

When statistically significant gender differences in symptom endorsement were observed, women consistently reported higher rates, although the effect sizes were small (ranging from φ = .05 to .12). The largest differences were found for “Loses things” (endorsed by approximately 71% of men and 80% of women), “Feels restless” (78.5% of men vs. 88% of women), and “Talks excessively” (52% of men vs. 63% of women).

### 3.4 Childhood symptoms

Endorsement rates for childhood ADHD symptoms by gender are also detailed in Table 2. A similar pattern emerged as in adulthood, but with overall lower percentages. “Difficulty sustaining attention” remained the most commonly endorsed symptom at about 84%, while “Interrupts or intrudes on others” was the least endorsed at about 38%.

Gender differences in childhood symptom endorsement revealed a reversed trend compared to adulthood, with men being more likely to endorse most of the symptoms. The largest differences were found for “Talks excessively” (endorsed by approximately 42% of men and 55% of women), “Leaves seat in classroom” (46% of men vs. 35% of women), and “Avoids tasks that require sustained mental effort” (78% of men vs. 70% of women). All effect sizes were small (ranging from φ = .05 to.12).

### 3.5 Adulthood symptom examples

The endorsement rates for all symptom examples by gender are provided in the Supplementary Material. Most adulthood examples had high endorsement rates, with the ten most frequently endorsed examples listed in Table 3. Examples endorsed by fewer than 25% of patients are shown in Table 4. Since these examples appear not to align well with the typical presentation of ADHD, they could be replaced with more representative scenarios.

**Table 3:** Endorsement percentages for most endorsed examples, by gender (*n* = 2,257).

	Males	Females	χ^2^	φ	OR (95% CI)
Adulthood symptom examples					
Quickly distracted by own thoughts or associations	82.86	84.30	1.07	0.02	0.90 (0.72–1.13)
Often postpone boring or difficult tasks	80.25	83.65	0.27	0.01	0.79 (0.64–0.99)
Difficulty shutting off from external stimuli	80.25	82.34	0.71	0.02	0.87 (0.70–1.08)
Easily distracted by noises or events	78.77	81.76	0.36	0.01	0.83 (0.67–1.02)
Not able to keep attention on tasks for long	78.09	82.05	0.12	0.01	0.78 (0.63–0.96)
Starts tasks but quickly loses focus and is easily sidetracked	74.35	82.34	0.54	0.02	0.62 (0.51–0.76)
Does things that are muddled up together without completing them	74.12	79.14	0.00	0.00	0.75 (0.62–0.92)
Feeling restless or agitated inside	70.94	80.01	1.17	0.03	0.61 (0.50–0.74)
Easily distracted by unrelated thoughts	72.30	77.62	0.01	0.00	0.75 (0.62–0.91)
Needing a time limit to complete tasks	65.49	72.38	0.42	0.02	0.72 (0.60–0.87)
Childhood symptom examples					
Easily distracted by noises or events	75.82	72.82	0.01	0.00	1.17 (0.96–1.42)
In the classroom, often looking outside	70.83	65.41	0.43	0.02	1.28 (1.07–1.54)
Easily distracted	66.4	62.35	0.11	0.01	1.19 (1.00–1.42)
Difficulty concentrating	61.63	57.19	0.28	0.01	1.20 (1.01–1.43)
Difficulty keeping attention on schoolwork	59.02	55.74	0.04	0.01	1.14 (0.96–1.36)
Messy room/desk and/or work	51.76	60.03	11.05**	0.09	0.71 (0.60–0.85)
Dreamy or preoccupied	55.51	54.43	0.20	0.01	1.04 (0.88–1.24)
Difficulty remaining focused during lectures and/or conversations	50.74	49.64	0.15	0.01	1.05 (0.88–1.24)
Avoidance of homework or has an aversion to this	56.41	46.00	7.01**	0.08	1.52 (1.28–1.80)
Starts tasks but quickly loses focus and is easily sidetracked	47.33	49.78	2.38	0.05	0.91 (0.77–1.07)
Adulthood impairment examples					
Unable to relax properly during free time	53.01	68.02	10.5**	0.09	1.25 (1.04–1.51)
Perfectionism	50.17	63.37	8.25**	0.08	1.12 (0.92–1.36)
Difficulty with administrative work/planning	52.55	50.44	3.20	0.05	0.90 (0.74–1.09)
Uncertainty through negative comments of others	44.95	53.92	3.48	0.06	1.02 (0.83–1.27)
Difficultly maintaining social contacts	47.45	52.03	0.18	0.01	1.09 (0.92–1.29)
Negative self-image due to experiences of failure	46.42	51.89	0.53	0.02	1.11 (0.80–1.55)
Difficulty with housekeeping and/or administration	45.52	47.67	0.10	0.01	1.05 (0.87–1.26)
Fear of failure in terms of starting new things	39.5	50.51	7.51**	0.08	1.66 (1.26–2.19)
Being continually busy and therefore becoming overtired	34.17	47.09	13.42***	0.12	0.87 (0.67–1.14)
Unable to finish a book or watch a film all the way through	38.59	41.93	0.06	0.01	1.25 (1.04–1.51)
Childhood impairment examples					
Difficulty doing homework	58.12	51.09	6.29*	0.07	1.33 (1.12–1.58)
Uncertainty through negative comments of others	43.13	52.69	8.40**	0.09	0.68 (0.57–0.81)
Negative self-image due to experiences of failure	36.66	42.73	3.89*	0.07	0.78 (0.65–0.92)
Fear of failure in terms of starting new things	32.12	42.73	13.87***	0.13	0.63 (0.53–0.76)
Perfectionism	29.17	44.33	29.57***	0.18	0.52 (0.43–0.62)
Comments from teachers about behaviour or concentration	43.36	33.28	16.58***	0.14	1.53 (1.29–1.83)
Lower educational level than expected based on IQ	37.91	30.96	9.05**	0.11	1.36 (1.14–1.63)
Being teased	28.83	32.41	1.63	0.05	0.84 (0.70–1.02)
Excessive intense reaction to criticism	25.99	33.21	7.99**	0.11	0.71 (0.59–0.85)
Unable to relax properly during free time	25.65	31.32	4.97*	0.09	1.33 (1.12–1.58)

* *p* < .05; **p < .01; ***p < .001. OR, odds ratio. OR > 1 indicates higher odds in males; OR < 1 indicates higher odds in females. The 95% Confidence Interval (CI) shows the range within which the true OR likely falls.

**Table 4:** Endorsement percentages for least endorsed examples, by gender (*n* = 2,257).

	Males	Females	χ^2^	φ	OR (95% CI)
Adulthood symptom examples					
Quickly distracted by own thoughts or associations	9.31	12.50	2.92	0.12	0.90 (0.72–1.13)
Often postpone boring or difficult tasks	14.19	13.01	1.72	0.07	0.79 (0.64–0.99)
Difficulty shutting off from external stimuli	14.30	14.17	0.43	0.04	0.87 (0.70–1.08)
Easily distracted by noises or events	11.80	16.13	4.32	0.11	0.83 (0.67–1.02)
Not able to keep attention on tasks for long	12.26	16.06	3.03	0.09	0.78 (0.63–0.96)
Starts tasks but quickly loses focus and is easily sidetracked	15.44	15.48	0.33	0.03	0.62 (0.51–0.76)
Does things that are muddled up together without completing them	16.91	17.51	1.06	0.04	0.75 (0.62–0.92)
Feeling restless or agitated inside	21.57	18.10	6.32	0.12	0.61 (0.50–0.74)
Easily distracted by unrelated thoughts	21.34	20.35	1.45	0.05	0.75 (0.62–0.91)
Needing a time limit to complete tasks	20.89	22.38	0.00	0.00	0.72 (0.60–0.87)
Too much time needed to complete detailed tasks	23.95	22.09	2.69	0.07	0.63 (0.53–0.76)
Work is inaccurate	26.90	21.29	11.87***	0.15	0.65 (0.54–0.78)
Forgets to do chores or run errands	27.13	21.22	12.87***	0.15	0.83 (0.69–0.98)
Talks during activities when this is not appropriate	20.54	25.58	2.81	0.07	0.96 (0.81–1.14)
Childhood symptom examples					
Fails to meet deadlines	10.78	9.08	0.86	0.06	1.21 (0.91–1.60)
Starts using people's things without asking or permission	10.67	11.26	0.58	0.05	0.94 (0.72–1.23)
Difficulty keeping himself/herself entertained	11.01	11.19	0.23	0.03	0.98 (0.75–1.29)
Not giving others room during a conversation	9.53	12.28	5.04*	0.14	0.75 (0.57–0.99)
Crosses the road without looking	12.15	11.48	0.01	0.01	1.07 (0.82–1.38)
Gets in a panic if other people move things around	11.35	14.03	4.38*	0.12	0.78 (0.61–1.02)
Able to control restlessness, but feels stressed as a result	15.32	13.52	0.50	0.04	1.16 (0.91–1.47)
Coming across as being tactless	15.10	14.24	0.01	0.01	1.07 (0.84–1.36)
Difficultly playing alone	16.00	13.81	0.85	0.05	1.19 (0.94–1.51)
Unable to watch TV or films quietly	14.30	15.92	0.95	0.04	0.88 (0.70–1.12)
Not checking the answers in homework	20.89	16.06	6.49*	0.11	1.38 (1.11–1.71)
Interrupts the games or activities of others	18.27	17.81	0.04	0.01	1.03 (0.83–1.29)
Being punished for talking too much	16.80	19.04	2.76	0.08	0.86 (0.69–1.07)
Leaves the reverse side of a test unanswered	19.64	18.39	0.04	0.01	1.08 (0.87–1.34)
Others find you restless or difficult to keep up with	19.98	18.53	0.09	0.01	1.10 (0.89–1.36)
Forgets to do chores or run errands	21.11	18.75	0.58	0.04	1.16 (0.94–1.43)
Always being the first to talk or act	20.43	19.62	0.00	0.00	1.05 (0.85–1.30)
Adulthood impairment examples					
Contact with the police/the courts	6.58	1.31	48.94***	0.80	5.32 (3.11–9.09)
Divorced owing to symptoms	4.77	3.92	1.61	0.13	1.23 (0.81–1.85)
Accidents/loss of driving licence as a result of reckless driving behaviour	6.36	2.91	17.83***	0.43	2.27 (1.50–3.43)
Problems with sexuality as a result of symptoms	5.79	7.27	0.88	0.08	0.78 (0.55–1.11)
Not achieving promotions	7.38	6.69	1.03	0.08	1.11 (0.80–1.55)
Injuries as a result of excessive sport	10.44	5.60	20.76***	0.35	1.97 (1.44–2.70)
Not daring to start a relationship	8.74	8.21	0.76	0.06	1.07 (0.79–1.45)
Problems with upbringing as a result of symptoms	9.53	8.50	1.60	0.09	1.13 (0.85–1.52)
Left work following arguments or dismissal	12.94	8.21	15.74***	0.26	1.66 (1.26–2.19)
Financial problems or gambling	14.30	9.59	14.22***	0.23	1.57 (1.21–2.04)
Sickness benefits/disability benefit as a result of symptoms	10.90	12.28	0.18	0.03	0.87 (0.67–1.14)
Unequal partner relationship owing to symptoms	14.98	13.23	2.79	0.09	1.16 (0.91–1.47)
Childhood impairment examples					
Contact with the police/courts	6.36	1.74	33.19***	0.64	3.82 (2.35–6.22)
Followed special education on account of symptoms	7.49	2.91	24.96***	0.49	2.70 (1.81–4.04)
Injuries as a result of excessive sport	7.60	5.16	5.72*	0.20	1.51 (1.07–2.14)
Being a bully	9.53	5.23	15.22***	0.31	1.91 (1.38–2.65)
Little contact with family on account of conflicts	7.95	9.52	4.20	0.08	0.82 (0.61–1.11)
Lower educational level than expected based on IQ	10.90	12.06	0.44	0.04	0.89 (0.68–1.16)
Increased number of accidents	13.39	10.83	3.48	0.11	1.27 (0.98–1.65)
Conflicts as a result of communication problems	11.46	13.88	2.02	0.08	0.80 (0.62–1.04)
Sensation seeking and/or taking too many risks	16.57	11.34	12.01***	0.20	1.55 (1.22–1.98)
Low self-assertiveness as a result of negative experiences	12.03	16.79	7.33**	0.15	0.68 (0.53–0.87)

* *p* < .05; **p < .01; ***p < .001. OR, odds ratio. OR > 1 indicates higher odds in males; OR < 1 indicates higher odds in females. The 95% Confidence Interval (CI) shows the range within which the true OR likely falls.

Gender differences in example endorsement were statistically significant in several cases, though effect sizes were small. The largest differences were seen in “Rigid use of lists to make sure things aren’t forgotten”, endorsed by about 19.5% of men and 30% of women (φ = .17; see Supplementary Material) and “Forgets to do chores or run errands”, endorsed by 27% of men and 21% of women (φ = .16; see Table 3).

### 3.6 Childhood symptom examples

Childhood symptom examples were less frequently endorsed than adulthood examples. The most endorsed examples are shown in Table 3. Examples endorsed by fewer than 20% of patients are shown in Table 4. These examples potentially need revision to better fit typical ADHD presentations.

Gender differences in childhood example endorsement were slightly larger than in adults, although still small. The largest differences were seen in hyperactivity symptoms, where men more frequently endorsed examples from “Leaves seat” and “Feels restless”, while women more frequently endorsed examples from “Talks excessively”. Specifically, the largest discrepancies were seen in “Comments in school reports about talking too much”, endorsed by 17% of men and 28% of women (φ = .24; see Supplementary Material), and “Known as a chatterbox”, endorsed by 36.5% of men and 55% of women (φ = .21; see Supplementary Material).

### 3.7 Adulthood impairment

Table 5 shows the endorsement rates for each adulthood ADHD impairment category by gender. The most affected areas for both men and women were work/education and self-confidence, with women more frequently reporting self-confidence issues (endorsed by approximately 81% of men and 89% of women, φ = .12), while men more often reported relationship or family-related impairments (61% of men vs. 57% of women, φ = .04). Women also reported more frequent impairments in social contacts (57% of men vs. 62% of women, φ = .04) and free time (71% of men vs. 80% of women, φ = .10). All effect sizes were small.

**Table 5:** Endorsement percentages for adulthood and childhood impairment categories, by gender (*n* = 2,257).

	Adulthood impairment					Childhood impairment				
	Males	Females	χ^2^	φ	OR (95% CI)	Males	Females	χ^2^	φ	OR (95% CI)
Work/education	88.42	85.83	2.94	0.04	1.26 (0.98–1.63)	88.65	79.72	29.99***	0.12	1.99 (1.55–2.54)
Relationship/family	61.07	56.54	4.34*	0.04	1.21 (1.01–1.43)	44.04	41.86	0.96	0.02	1.09 (0.92–1.30)
Social contacts	57.21	61.56	4.05*	0.04	0.83 (0.70–0.99)	46.88	52.03	5.51*	0.05	0.81 (0.69–0.96)
Free time/hobby	70.83	79.94	24.23***	0.10	0.61 (0.50–0.74)	50.28	49.49	0.11	0.01	1.03 (0.87–1.22)
Self-confidence	80.59	89.03	30.56***	0.12	0.51 (0.40–0.65)	64.59	77.25	42.41***	0.14	0.54 (0.45–0.65)

* *p* < .05; ***p < .001. OR, odds ratio. OR > 1 indicates higher odds in males; OR < 1 indicates higher odds in females. The 95% Confidence Interval (CI) shows the range within which the true OR likely falls.

### 3.8 Childhood impairment

Childhood impairment endorsement rates by gender are presented in Table 5. The pattern mirrored adulthood, with work/education and self-confidence being the most commonly reported areas of difficulty. The largest gender difference was in self-confidence, with women more likely to report difficulties (65% of men vs. 77% of women, φ = .14), consistent with the adulthood findings. Other gender differences varied from those in adulthood: women were less likely than men to report difficulties in education (89% of men vs. 80% of women, φ = .12) and social contacts (47% of men vs. 52% of women, φ = .05), whereas there were no other differences in the remaining categories. Effect sizes were small.

### 3.9 Adulthood impairment examples

The endorsement rates for all impairment examples by gender are detailed in the Supplementary Material. Overall, these examples were less frequently endorsed than symptom examples, with most reported by 10%–50% of patients. Table 3 shows the most frequently endorsed examples, while examples endorsed by fewer than 15% of patients appear in Table 4. Many of the less-endorsed examples may not represent the majority of ADHD patients and might therefore benefit from replacement.

Gender differences were statistically significant across several impairment examples, with most differences showing small effect sizes. However, medium or even large differences were observed in several of the least commonly endorsed items in Table 4, particularly those related to impulsivity, such as car accidents and gambling, where men reported higher rates. Among more frequently endorsed items, the largest gender differences were in “Binge eating” (16.5% of men vs. 23% of women, φ = .13; see Supplementary Material), “Being continually busy and becoming overtired” (34% of men vs. 47% of women, φ = .12; see Table 3), and “Did not complete education/training needed for work” (30.5% of men vs. 26% of women, φ = .12; see Supplementary Material).

### 3.10 Childhood impairment examples

Childhood impairment examples were endorsed even less frequently than those for adulthood, with most items reported by only 10%–40% of patients. The most commonly endorsed examples are presented in Table 3, and those endorsed by fewer than 15% of patients can be found in Table 4. Again, these examples appear to describe uncommon situations that may be revised to better reflect typical patient experiences.

Statistically significant gender differences emerged in several childhood impairment examples. Similar to the patterns observed in adulthood, medium to large effect sizes were found among the least frequently endorsed items, as detailed in Table 4. Among the remaining items, the largest gender differences were found in “Education not completed/rejected from school”, endorsed by approximately 28% of men and 18% of women (φ = .23; see Supplementary Material), “Staying back (repeating classes) as a result of concentration problems”, endorsed by around 33% of men and 22% of women (φ = .21; see Supplementary Material), and “Perfectionism”, endorsed by about 29% of men and 44% of women (φ = .18; see Table 3).

## 4 Discussion

To better understand the manifestation of ADHD in adult men and women, this study examined endorsement rates in the DIVA-5 assessment in a large Dutch clinical sample. We focused on overall endorsement rates of ADHD symptoms and impairments, along with endorsement of specific examples provided in the interview. By investigating these patterns, we aimed to gain insight into how frequently each symptom and area of impairment was endorsed across the sample as a whole and to explore any gender differences that emerged in these endorsement frequencies.

### 4.1 Gender differences in endorsement frequencies

Our primary finding is that ADHD manifests in a remarkably similar way in men and women. This is consistent with previous research [see e.g., (26, 27)], suggesting that delays in diagnosis that have been observed in women may stem less from symptom differences and more from societal factors, such as clinical biases. For instance, clinicians may be less likely to recognise the same ADHD symptoms in women due to stereotypes that portray ADHD as a predominantly male condition (28). Additionally, women may display coping strategies, such as heightened organisational efforts or overcompensation in social settings, which can mask ADHD symptoms and further complicate recognition by clinicians (10). Nonetheless, while the gender differences observed in this clinical sample are small, they remain noteworthy, particularly given the likelihood that such differences are more pronounced in non-clinical populations, as discussed in Section 4.3.

Interestingly, where statistically significant gender differences in current symptom endorsement were observed, women consistently reported higher rates. This was not the case for other, comparable studies: for instance, Cortese et al. (16), examining a community sample of adults with ADHD in the United States, found men reporting higher rates of one inattention symptom and five hyperactivity/impulsivity symptoms. This discrepancy may stem from the current study's clinical sample. Research suggests that females often require more severe symptoms to be referred for ADHD assessment and treatment (29), which could increase symptom severity among women in clinical settings. However, this does not explain why no difference in overall ADHD severity was found between men and women in this study.

It is particularly noteworthy that women in this study had higher endorsement rates for six of the current hyperactivity/impulsivity symptoms, which contrasts with childhood symptom endorsements, where men had higher rates of three hyperactivity/impulsivity symptoms, while women had higher rates of only two. This shift might partly be explained by differences in the DIVA-5's symptom examples for different life stages. Childhood examples of hyperactivity typically involve overt behaviours, such as “Always running around where it is inappropriate” or “Being told to remain seated”, which men endorsed at higher rates. In contrast, adult examples focus on more internalised or less visible behaviours, such as “Stepping over own boundaries” or “Fiddling with hair or biting nails”, which were more frequently endorsed by women. This pattern might arise from the fact that girls are generally socialised to act more “maturely” (30), so they may express hyperactivity in subtler, less noticeable ways during childhood, making them less likely to fit conventional diagnostic criteria that emphasise externalised behaviours. By adulthood, when expectations around hyperactivity shift to encompass internal experiences, women's expressions of hyperactivity may align more closely with the diagnostic criteria, leading to similar endorsement rates across genders. This may help explain why the gender gap in ADHD prevalence appears to narrow in adulthood (31).

However, this does not explain why we observed a higher rate of hyperactivity symptoms in women, which runs counter to the prevailing view that the opposite is true (10, 27, 31, 32). A closer look at the literature reveals that much of the evidence supporting this view comes from studies focused on children rather than adults. By analysing both current and retrospective symptoms, our study reveals that gender patterns in hyperactivity may evolve across the lifespan. Our results suggests that developmental differences may underlie these gender disparities, perhaps due to socialisation, coping strategies, or physiological changes across the lifespan [see e.g., (33)].

To our knowledge, the only other study that found higher hyperactivity rates in women is Fedele et al. (34), who speculated that women may report higher hyperactivity on self-report measures due to holding themselves to stricter standards. In our study, however, the DIVA-5 was administered by clinicians, usually in the presence of a family member or partner, which likely reduces the influence of self-report bias. This provides a more robust indication that in clinical settings, women might indeed display slightly higher rates of hyperactivity in adulthood than men, though the differences are small and may go undetected in studies with smaller samples [e.g., (26)]. Notably, the largest gender differences in our study were seen in hyperactivity symptoms related to talkativeness, which women endorsed more frequently in both childhood and adulthood. This aligns with previous research (26, 35), suggesting that verbal hyperactivity may be a particularly relevant marker for identifying ADHD in women.

Another intriguing finding in our study is that male patients reported more childhood inattention symptoms than female patients. This finding diverges from previous studies, which show either higher rates of inattention among girls (34, 36) or similar rates across genders (37). These studies used current, rather than retrospective, reports of childhood ADHD symptoms. Retrospective recall can be inconsistent, with some studies indicating adults may overestimate (38) or underestimate (39), childhood symptoms, meaning that the retrospective nature of our study may impact accuracy. Additionally, the types of examples provided for childhood inattention in the DIVA-5 might influence endorsement rates. Several of the DIVA-5 examples reflect negative classroom-based behaviours that tend to be more common among boys (40), such as “Others comment about careless work”, and “Not completing homework or handing it in”. This may bias symptom endorsement toward male patients, as we observed that they more frequently endorsed these examples. Including examples more representative of girls' experiences with inattention might balance this gender discrepancy.

In terms of example endorsement for current inattention symptoms, another notable pattern emerged: the largest differences in selection frequency involved men endorsing examples that reflected difficulties in daily life, while women more frequently selected examples that reflected coping mechanisms. For instance, men more often endorsed examples such as “Work is inaccurate”, “Fails to meet deadlines”, and “Forgets to do chores or run errands”, whereas women more often endorsed examples like “Inflexible because of the need to keep to schedules”, “Creating schedules but not using them”, and “Rigid use of lists to make sure things aren’t forgotten”. This pattern aligns with the theory that women with ADHD are better at developing coping strategies that mask underachievement and performance issues (10).

When examining the endorsement of overall impairment categories for both adulthood and childhood, we found only minor gender differences. The largest difference was in self-confidence, with women consistently reporting higher levels of impairment. This is in line with theories suggesting that ADHD symptoms conflict with societal expectations of femininity, potentially leading to struggles with self-esteem and self-confidence among women with ADHD (41). However, a recent review by Pedersen et al. (42) found no gender differences in self-esteem among people with ADHD. This discrepancy may be explained by the small sample sizes in the studies included in the review (43, 44) which may not have been large enough to detect the subtle effect we observed.

Overall, it is important to note that nearly all the gender differences we identified were associated with small effect sizes. While these differences are statistically significant, their clinical meaningfulness remains uncertain, as effect sizes of this magnitude typically fall below established benchmarks for practical significance (45). It is therefore unlikely that these differences have substantial real-world or clinical implications. Importantly, the goal of this exploratory study is not to provide clinical recommendations but rather to generate hypotheses for future research. Further investigation is needed to determine whether and how ADHD manifestations and diagnostic processes may differ meaningfully between genders.

### 4.2 Overall endorsement frequencies

Overall, symptom and symptom example endorsement rates across childhood and adulthood suggest that the DIVA-5 broadly captures ADHD symptomatology. All core symptoms and most examples were endorsed by more than a third of participants in both genders, suggesting that the tool captures essential ADHD traits effectively. Impairment categories were also widely endorsed, with even the least frequently endorsed categories marked by about half of patients. This underscores the substantial impact of ADHD on the average patient, particularly in the work/education domain, which was endorsed by eighty to ninety percent of patients in both adulthood and childhood, aligning with previous research (24). Given that the impairments associated with ADHD can be diminished with psychological and pharmacological treatment [for reviews, see (46), and (47)] our findings highlight the importance of addressing and treating the disorder.

We also observed that impairment examples were endorsed at much lower rates than symptom examples. Several examples, such as losing one's driver's license or having contact with the police due to ADHD symptoms, appear to be less representative of typical patient experiences in this clinical sample. This is noteworthy because ADHD is associated with considerable stigma, with ADHD patients sometimes being perceived as “violent” (48) or even “dangerous” (49), leading to potential social consequences, such as difficulty renting housing or receiving job recommendations (50). This stigma may arise from an overestimation of the risk of harmful behaviour among those with ADHD. However, in this study, the most frequently endorsed impairment examples were related to internalised struggles, such as perfectionism or internal tension. Recognising the prevalence of these internalised impairments over disruptive externalised impairments could help challenge misconceptions and reduce stigma by aligning diagnostic tools more closely with the lived experiences of people with ADHD. Moreover, this approach would better reflect symptom manifestations in women, who were less likely to endorse the more extreme externalised impairment examples in our study.

### 4.3 Strengths, limitations and future directions

The main strength of this study lies in its scale and focus, making it one of the largest investigations of overall endorsement rates for ADHD symptoms and impairments, as well as gender differences in these domains, to date. By analysing both specific behavioural examples and broader categories of symptoms and impairment, the study offers a detailed exploration of ADHD that can be used for hypotheses generating purposes in many different contexts. Moreover, this is the first in-depth analysis of patient responses to the widely used DIVA-5, providing practical insights for future studies.

An important limitation in our study is the measurement of gender. Although transgender, nonbinary, and gender-diverse people are overrepresented in neurodiverse populations (51), they remain largely excluded from research samples. This study is no exception: at the time of data collection, the registration procedure at the Parnassia Groep allowed only male or female entries based on patients' legal gender. This introduces two issues. Firstly, potential nonbinary participants are likely misclassified into categories that do not align with their identity. Secondly, some participants' legal gender may not match their gender identity. Although it is possible to change one's legal gender in the Netherlands, some people may choose not to or may delay this change due to reasons like administrative hurdles or societal stigma (52). Given that around 1.3% of the Dutch population identifies as transgender, gender-diverse, or intersex (57), we would expect around 30 individuals in our sample to belong to these groups—a conservative estimate given the overrepresentation of these populations among those with ADHD. Unfortunately, these individuals were not sufficiently accounted for in our analysis. It is essential that future studies address this gap.

Including diverse gender identities in research is not only important ethically but may also help to disentangle the effects of sex and gender on ADHD symptomatology. Some observed differences in ADHD symptoms may be biologically based; for example, many people report symptom fluctuations that correlate with hormonal changes over the menstrual cycle (53). Other differences may arise from societal expectations associated with gender roles. For instance, rambunctious behaviour in boys is more tolerated, which may lead girls to internalise symptoms more readily to align with gendered behavioural norms (54). As such, ADHD symptoms may be influenced by the effects of sex, gender, or a combination of both. Including samples more diverse in terms of gender and sex in future research could help to untangle these interactions.

Another limitation of this study is referral bias. Because participants were drawn from clinical settings, the sample likely consists of people whose symptoms were pronounced or disruptive enough to lead to a formal referral. This could skew findings toward more severe or “typical” presentations of ADHD, which may not represent the broader spectrum of symptoms across genders. To mitigate referral bias, future research could incorporate population-based approaches that might provide a more comprehensive view of ADHD symptomatology. Such research would also enable comparisons of DIVA-5 responses between people with and without ADHD, which would help to clarify its diagnostic specificity. If certain examples are similarly endorsed by both groups, they may lack the discriminatory power needed for accurate diagnosis and should be replaced by examples more distinct to ADHD.

Moreover, examining gender differences in populations with and without ADHD could deepen our understanding of how ADHD manifests across genders. While our findings indicate that the gender differences we observed may be too subtle to be clinically relevant, it is important to consider that our sample only includes patients who were diagnosed with ADHD. If we assume that some women with ADHD are being overlooked due to their symptom presentation, it follows that they are not represented in our sample. Therefore, future research should also explore subclinical samples, where we hypothesise that gender differences would be more pronounced.

Finally, it is essential to remember that this was an exploratory study aimed at generating hypotheses for future research, and the *p*-values presented here are not intended to be interpreted in the conventional manner. Since *p*-values are designed for testing predefined hypotheses, their use in this context is more indicative than confirmatory and should be approached with caution, especially in cases with small effect sizes.

Our research group is aiming to develop a new edition of the DIVA-5 designed to better capture ADHD manifestations in women and girls. We plan to use focus groups and a Delphi study involving researchers, clinicians, and patients to identify symptom and impairment examples that potentially better capture the female phenotype of ADHD. To accommodate these new examples, we intend to remove those identified in this study as least frequently endorsed, as they do not appear to align with most patients' experiences. The original examples in the DIVA-5 were selected based on clinical expertise rather than empirical data, so grounding our revisions in endorsement rates will hopefully enhance both diagnostic accuracy and relevance.

## 5 Conclusion

This study examined DIVA-5 data from a large sample of patients, focusing on overall endorsement rates of ADHD symptoms, impairments, and specific examples of both, with particular attention to gender differences. The results indicate that ADHD presents in largely similar ways in men and women, with only small but notable differences observed. This aligns with most previous research that suggests gender-based variations in ADHD manifestation tend to be modest. We also discussed potential measurement issues that may have influenced these results. To support future research into ADHD symptomatology, we are providing the aggregated endorsement rates for our sample in our Supplementary Material. These results include information beyond the specific findings presented here, offering opportunities for additional insights into ADHD presentation. We encourage other researchers to build on these findings for a deeper understanding of ADHD presentations across genders.

## Data Availability

The data analyzed in this study is subject to the following licenses/restrictions: The dataset analysed for this study is available upon request, and we are working towards making it openly accessible to promote transparency and further research, though this is currently not feasible due to privacy constraints. Requests to access these datasets should be directed to Noemi Platania, n.platania@parnassiagroep.nl.
